# Prevalence and clinical characteristics of Danon disease among patients with left ventricular hypertrophy and concomitant electrocardiographic preexcitation

**DOI:** 10.1002/mgg3.638

**Published:** 2019-03-30

**Authors:** Yang Liu, Xin Chen, Feng Wang, Yingcong Liang, Hai Deng, Hongtao Liao, Qianhuan Zhang, Bin Zhang, Xianzhang Zhan, Xianhong Fang, Michael Shehata, Xunzhang Wang, Yumei Xue, Shulin Wu

**Affiliations:** ^1^ Guangdong Cardiovascular Institute, Guangdong General Hospital, Guangdong Academy of Medical Sciences Guangzhou China; ^2^ Guangdong Provincial Key Laboratory of Clinical Pharmacology Guangdong General Hospital, Guangdong Academy of Medical Sciences Guangzhou China; ^3^ Department of Geriatrics Guangdong General Hospital, Guangdong Academy of Medical Sciences Guangzhou China; ^4^ Heart Institute, Cedars Sinai Medical Center Los Angeles California

**Keywords:** Danon disease, *LAMP2*, left ventricular hypertrophy, ventricular preexcitation

## Abstract

**Background:**

Cardiac involvement in Danon disease typically manifests as left ventricular hypertrophy (LVH) and ventricular preexcitation. This study aimed to identify patients with Danon disease among patients with LVH and concurrent electrocardiographic preexcitation.

**Methods:**

Electrocardiographic preexcitation was identified in 10 of 197 patients with unexplained LVH in whom genetic testing was performed using next‐generation sequencing.

**Results:**

Three (3/10, 30%) patients with Danon disease were found in association with different mutations in the gene of lysosome‐associated membrane protein 2 (*LAMP2*). Compared to seven patients without Danon disease, these three patients presented with distinctive clinical phenotypes, including onset at an earlier age (20 ± 2 years vs. 53 ± 9 years, *p* < 0.001), more neurological involvements (100% vs. 0, *p* = 0.008), higher electrocardiographic voltages (10 ± 1 mV vs. 5 ± 1 mV, *p* < 0.001), wider QRS complexes (163 ± 5 ms vs. 115 ± 20 ms, *p* = 0.006), less common asymmetric hypertrophy (0% vs. 86%, *p* = 0.033), and more frequent elevation of three serum enzymes (creatine kinase, aspartate aminotransferase, and lactate dehydrogenase). Intracellular vacuoles accumulation with deficiencies of *LAMP2* protein was found in both cardiac and skeletal myocytes of patients with Danon disease.

**Conclusion:**

In patients with coexistent LVH and ventricular preexcitation, Danon disease is common with distinctive clinical presentations. Comprehensive assessment of these resemble patients can provide valuable findings for early identification and clinical decision making of patients with Danon disease.

## 1 INTRODUCTION

Danon disease, caused by the mutations in the gene of lysosome‐associated membrane protein 2 (*LAMP2*,OMIM*309060), is an X‐linked dominant disorder classically characterized by the triad of cardiomyopathy, skeletal myopathy, and intellectual disability (Danon et al., 1981; Nishino et al., 2000; Tanaka et al., 2000). Cardiomyopathy typically manifests a hypertrophic phenotype; ventricular preexcitation is the most frequently encountered ECG abnormality in such patients (D'souza et al., 2014). The pathological hallmark of Danon disease is intracytoplasmic vacuole accumulation containing glycogen granules and autophagic materials which lead to cardiac hypertrophy (Arad et al., 2005; Cheng et al., 2012; Nishino et al., 2000). Because of similar echocardiographic features, Danon disease could be misdiagnosed as hypertrophic cardiomyopathy (HCM) caused by genetic defects in cardiac structural proteins. Unlike HCM, however, individuals with Danon disease frequently experience rapid clinical deterioration from no or little symptoms and preserved systolic function to end‐stage heart failure (Maron et al., 2009). It is therefore important to distinguish myocardial hypertrophy associated with *LAMP2* mutations from that due to other disease‐causing genetic defects. The objective of this study was to identify patients with Danon disease among patients with coexistent unexplained left ventricular hypertrophy (LVH) and electrocardiographic preexcitation, and compare the clinical, electrocardiographic, and echocardiographic features of patients with or without Danon disease.

## 2 METHODS

### 2.1 Ethical compliance

The investigation was approved by the ethics committees of Guangdong General Hospital and performed in accordance with the declaration of Helsinki. An informed and written consent was obtained from all study subjects.

### 2.2 Study subjects

A total of 197 patients with unexplained LVH were enrolled between October 2015 and January 2018 at Guangdong General Hospital, Guangzhou, China (Table S1). All patients were evaluated on the basis of medical history, physical examination, 12‐lead ECG, and transthoracic echocardiography. LVH was defined by a wall thickness of at least 15 mm in one or more left ventricular myocardial segments on echocardiography (Elliott et al., 2014). Asymmetric hypertrophy indicated LVH with ratio between maximal thickness of interventricular septum (IVS) and left ventricular posterior wall (LVPW) more than 1.3. Ventricular preexcitation was typically characterized by short PR intervals (PR < 120 ms), initial QRS slurring (delta wave), or both during sinus rhythm. In standard 12‐lead ECG, left ventricular voltage was reported as the maximal S wave in V1 or V2 plus maximal R wave in V5 or V6 (S_V1_ or S_V2_ + R_V5_ or R_V6_). When available, pathological specimens were examined.

## 3 MOLECULAR GENETIC ANALYSIS

Ten patients with coexistent LVH and ventricular preexcitation underwent genetic testing to identify Danon disease. Genomic DNA was extracted from peripheral blood lymphocytes using a commercial kit (QIAGEN Co., Ltd, CA) for genetic screenings with next‐generation sequencing (KingMed Diagnostics, Guangzhou, China). The MiSeq Reagent Kit v3 (Illumina, San Diego, CA) was used to prepare DNA libraries. Targeted enrichment was performed using the TruSight One Sequencing Panel on the MiSeq platform (Illumina, San Diego, CA) which was designed to cover 12 Mb of genomic content with up to 62,000 exons in 4,813 clinical disease‐associated genes. In addition to comprehensive coverage of the major exon regions, the panel provided coverage of exon‐flanking regions as well. Genomic targets were determined based on information in the Human Gene Mutation Database (HGMD), the Online Mendelian Inheritance in Man (OMIM), the GeneTests, Illumina TruSight sequencing panels, and other available sequencing panels. The full list of these genes is available online (www.illumina.com/content/dam/illumina-marketing/documents/products/gene_lists/gene_list_trusight_one.zip).

All sequencing data was processed with Burrows–Wheeler Aligner for alignment with a reference sequence of hg19 and Genome Analysis Toolkit for variant calling. The ANNOVAR tool was used to analyze Variant Call format files and annotate potential variants. ANNOVAR utilized annotation databases including the 1,000 Genomes Project, dbSNP, ClinVar database, Polyphen‐2, and SIFT.

Candidate variants identified by the next‐generation sequencing were further validated by direct Sanger sequencing using an ABI 3500 Dx Genetic Analyzer (Applied Biosystems, Foster City, CA). The sequences of the wild‐type *LAMP2* gene were obtained from the National Center for Biotechnology Information protein database (GenBank Accession Number, RefSeq NM_002294.2).

### 3.1 Histological and immunofluorescent analysis

Specimens of muscle were biopsied from the right IVS via the right internal jugular vein, the quadriceps femoris muscle, or both. Specimens were fixed either in formalin for staining with hematoxylin and eosin and Masson's trichrome, and *LAMP2* immunofluorescent analysis or in glutaraldehyde for transmission electron microscopy (JEM1400‐Plus, JEOL Ltd., Tokyo, Japan). Immunofluorescent staining of *LAMP2 *was performed with a mouse monoclonal antibody (H4B4, ab25631, abcam, Cambridge, UK).

### 3.2 Statistical analysis

Continuous variables were reported as mean ± standard deviation or median and minimum and maximum values, and comparisons between groups were based on an unpaired Student's *t* test (parametric) or Mann–Whitney *U* test (non‐parametric), as appropriate. A Shapiro–Wilk test was carried out to determine normality of data distribution and variance homogeneity. Categorical variables were summarized as counts and percentages and compared using a Fisher's exact test. The significance was defined as a *p* < 0.05.

## 4 RESULTS

### 4.1 Clinical findings

Ventricular preexcitation was present in 10 (10/197, 5%) unrelated patients with unexplained LVH in whom genetic testing identified three (3/10, 30%) Danon disease patients with different *LAMP2 *mutations. In the remaining seven patients, one mutation in the gene encoding α‐galactosidase A (Fabry's disease), two in beta‐myosin heavy chain (*MYH7)*, one in alpha‐tropomyosin (*TPM1)*, and 1variant with unknown clinical significance in the gene encoding titin were, respectively, found in five patients, and the test result was negative in two patients. In this group, nobody had double or more genetic abnormalities. Of the seven patients without Danon disease, delta wave was positive in leads I and aVF and negative in lead V1 in three patients indicating a probable right anterosepal location of accessory pathways, and initial R wave in lead V1 was present in the remaining four patients indicating left‐side location (Figure S1).

**Figure 1:**
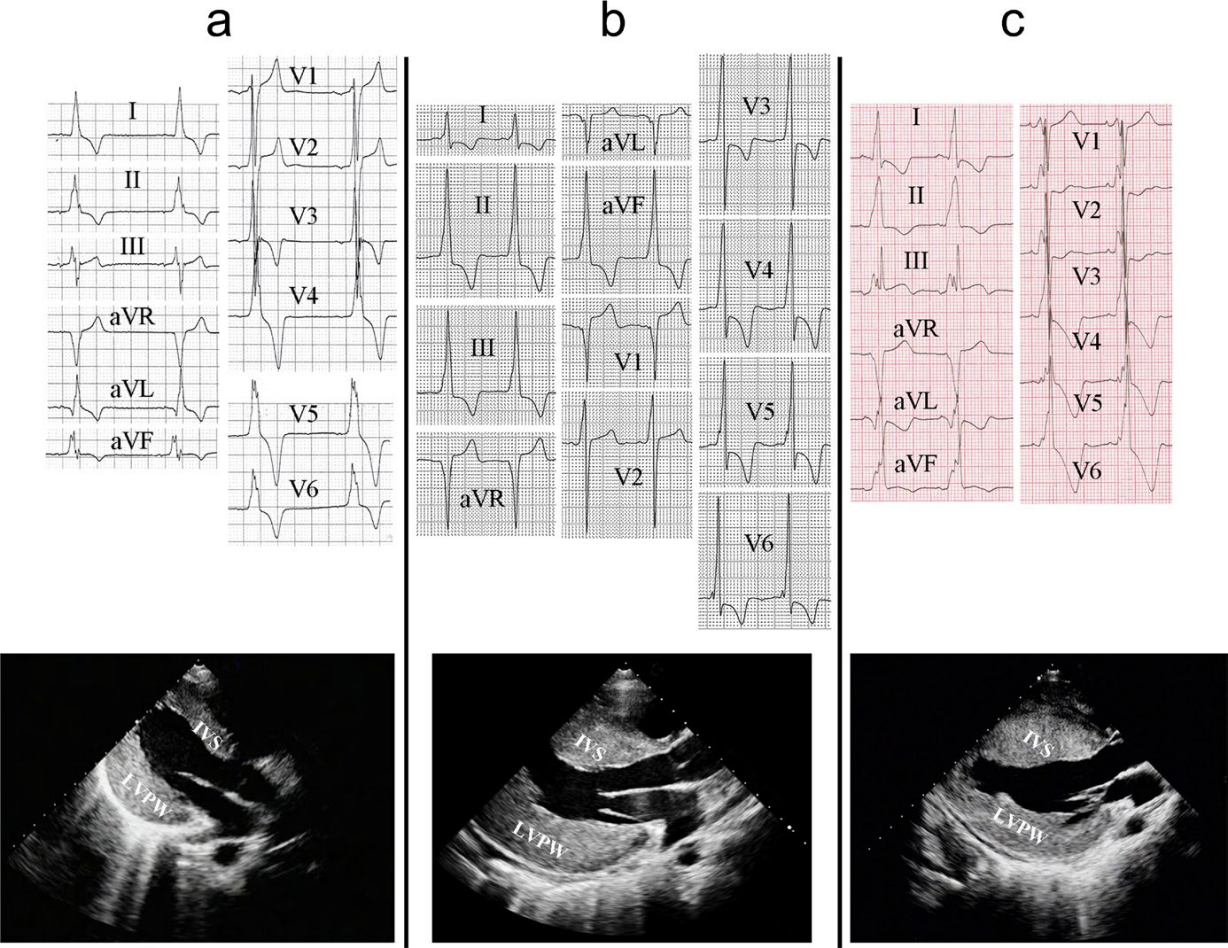
Surface ECGs (25 mm/s, 5 mm/mV) and parasternal long‐axis echocardiogram views of three patients with Danon disease. (a) Resting ECG demonstrates sinus bradycardia (50 bpm), normal PR interval (150 ms) but with positive delta waves in leads I, II, III, V1, and V6, high voltage of the left ventricle and inverted T waves in patient 1. Echocardiogram demonstrates concentric LVH with an IVS thickness of 19 mm and a LVPW thickness of 24 mm. (b) Resting ECG demonstrates sinus rhythm, a short PR interval (95 ms) with positive delta waves in leads I, II, III, aVF, V3‐–V6, as well as negative deltas in leads aVL and V1, high voltage of the left ventricle and inverted T waves in patient 2. Echocardiogram demonstrates concentric LVH with an IVS thickness of 33 mm and a LVPW thickness of 31 mm. (c) Resting ECG demonstrates sinus rhythm, a PR interval of 120 ms with positive delta waves in leads I, II, III, aVF, and V2–V6 as well as biphasic (±) delta waves in lead V1, high voltage of the left ventricle and inverted T waves in patient 3. Echocardiogram demonstrates concentric LVH with an IVS thickness of 29 mm and a LVPW thickness of 23 mm. IVS: interventricular septum; LVH: left ventricular hypertrophy; LVPW: left ventricular posterior wall

Of the three patients with Danon disease, all were male aged 17–23 years at diagnosis. The initial presentations at admission were variable, including exertional dyspnea, syncope, chest pain on exertion, and aborted cardiac arrest. They all had mild intellectual disability, mainly causing learning disorders, but only one patient had complaints of muscle weakness. Surface 12‐lead ECGs were strikingly abnormal, with short PR intervals, delta waves, or both and high voltage consistent with ventricular preexcitation; transthoracic echocardiography showed concentric LVH (Figure 1). Additionally, patient 1 experienced episodes of AV block with the longest R–R interval of 3.26 s; patient 3 presented with spontaneous ventricular fibrillation on ambulatory ECG monitoring during an aborted cardiac arrest. Laboratory abnormalities were present in all three patients and included significant elevation of N‐terminal pro‐brain natriuretic peptide (14‐ to 89‐fold over upper limit of normal range), creatine kinase (6‐ to 8‐fold), aspartate aminotransferase (9‐ to 14‐fold), lactate dehydrogenase (5‐ to 6‐fold), and cardiac troponin T (14‐ to 377‐fold). Individual clinical details of the three patients with Danon disease are summarized in Table 1.

**Table 1:** Detailed clinical characteristics of the three patients with Danon disease

	Patient 1		Patient 2	Patient 3	Normal range
Age, years	19		23	17	
Gender	M		M	M	
Chief complaint	Exertional dyspnea, syncope		Chest pain	Aborted cardiac arrest	
Intellectual disability	Mild		Mild	Mild	
Muscle weakness	No		No	Yes	
Electrocardiography					
Family history	Yes (sudden death)		Yes (WPW)	No	
S_V1_ or S_V2_ + R_V5_ or R_V6, _mV	9		11	10	
QRS width, ms	160		160	170	
Sinus bradycardia	Yes		No	No	
Intermittent AVB	Yes		No	No	
Ventricular fibrillation	No		No	Yes	
Echocardiography					
LA size, mm	33		39	30	
LV size, mm	41		32	42	
Maximal IVS thickness, mm	19		33	29	
Maximal LVPW thickness, mm	24		31	23	
LVEF, %	61		62	45	
NT‐proBNP, pg/ml	1,986		1,738	11,182	0–125
Serum enzymes					
CK, U/L		1,352	1,122	1,298	38–174
CK‐MB, U/L		17	169	43	0–24
AST, U/L		461	546	349	15–40
ALT, U/L		301	62	254	9–50
LDH, U/L		1,174	1,582	1,406	109–245
cTnT, pg/ml		190	5,272	339	0–14

Note

ALT: alanine aminotransferase; AST: aspartate aminotransferase; AVB: atrioventricular block; CK: creatine kinase; cTnT: cardiac troponin T; IVS: interventricular septum; LA: left atrium; LDH: lactate dehydrogenase; LV: left ventricle; LVEF: left ventricular ejection fraction; LVPW: left ventricular posterior wall; NT‐proBNP: N‐terminal pro‐brain natriuretic peptide; WPW: Wolff–Parkinson–White syndrome.

Compared to the remaining seven patients without Danon disease, the patients with Danon disease were younger (20 ± 2 years vs. 53 ± 9 years, *p* < 0.001) and more likely to have symptoms of intellectual disability (100% vs. 0, *p* = 0.008), and presented with higher left ventricular voltage (10 ± 1 mV vs. 5 ± 1 mV, *p* < 0.001), wider QRS complexes (163 ± 5 ms vs. 115 ± 20 ms, *p* = 0.006), and more prominent increase in LVPW thickness (26 ± 4 mm vs. 11 ± 2 mm, *p* < 0.001). Asymmetric hypertrophy was less common in patients with Danon disease than non‐Danon disease patients (0% vs. 86%, *p* = 0.033). Additionally, the occurrence of creatine kinase, aspartate aminotransferase, and lactate dehydrogenase more than threefold the upper limit of normal range was more common in patients with Danon disease than non‐Danon disease patients. The baseline characteristics of patients with and without Danon disease are summarized and compared in Table 2.

**Table 2:** Clinical characteristics of patients with and without Danon disease

	Danon disease (*n* = 3)	Non‐Danon disease (*n* = 7)	*p* value
Age at diagnosis, years	20 ± 2	53 ± 9	<0.001
Male gender	3 (100%)	7 (100%)	
Intellectual disability	3 (100%)	0	0.008
Electrocardiography			
S_V1_ or S_V2_ + R_V5_ or R_V6_, mV	10 ± 1	5 ± 1	<0.001
QRS duration, ms	163 ± 5	115 ± 20	0.006
Echocardiography			
LA size, mm	34 ± 4	40 ± 6	0.150
LV size, mm	38 ± 4	44 ± 4	0.096
Maximal IVS thickness, mm	27 ± 6	19 ± 4	0.057
Maximal LVPW thickness, mm	26 ± 4	11 ± 2	<0.001
Asymmetric hypertrophy	0	6 (86%)	0.033
LVEF, %	56 ± 8	62 ± 6	0.249
LVOTO	0	4 (57%)	0.200
NT‐proBNP > 3 times of ULN	3 (100%)	3 (50%)^a^	0.464
Serum enzymes			
CK > 3‐fold ULN	3 (100%)	0	0.008
CK‐MB > 3‐fold ULN	1 (33%)	0	0.300
AST > 3‐fold ULN	3 (100%)	0	0.008
ALT > 3‐fold ULN	2 (67%)	0	0.067
LDH > 3‐fold ULN	3 (100%)	0^a^	0.012
cTnT > 3‐fold ULN	3 (100%)	2 (33%)^a^	0.167

Note

ALT: alanine aminotransferase; AST: aspartate aminotransferase; CK: creatine kinase; cTnT: cardiac troponin T; IVS: interventricular septum; LA: left atrium; LDH: lactate dehydrogenase; LV: left ventricle; LVEF: left ventricular ejection fraction; LVOTO: left ventricular outflow tract obstruction; LVPW: left ventricular posterior wall; NT‐proBNP: N‐terminal pro‐brain natriuretic peptide.

a This value was not available in one patient. ULN: upper limit of normal range.

### 4.2 Molecular genetic analysis

**Figure 2:**
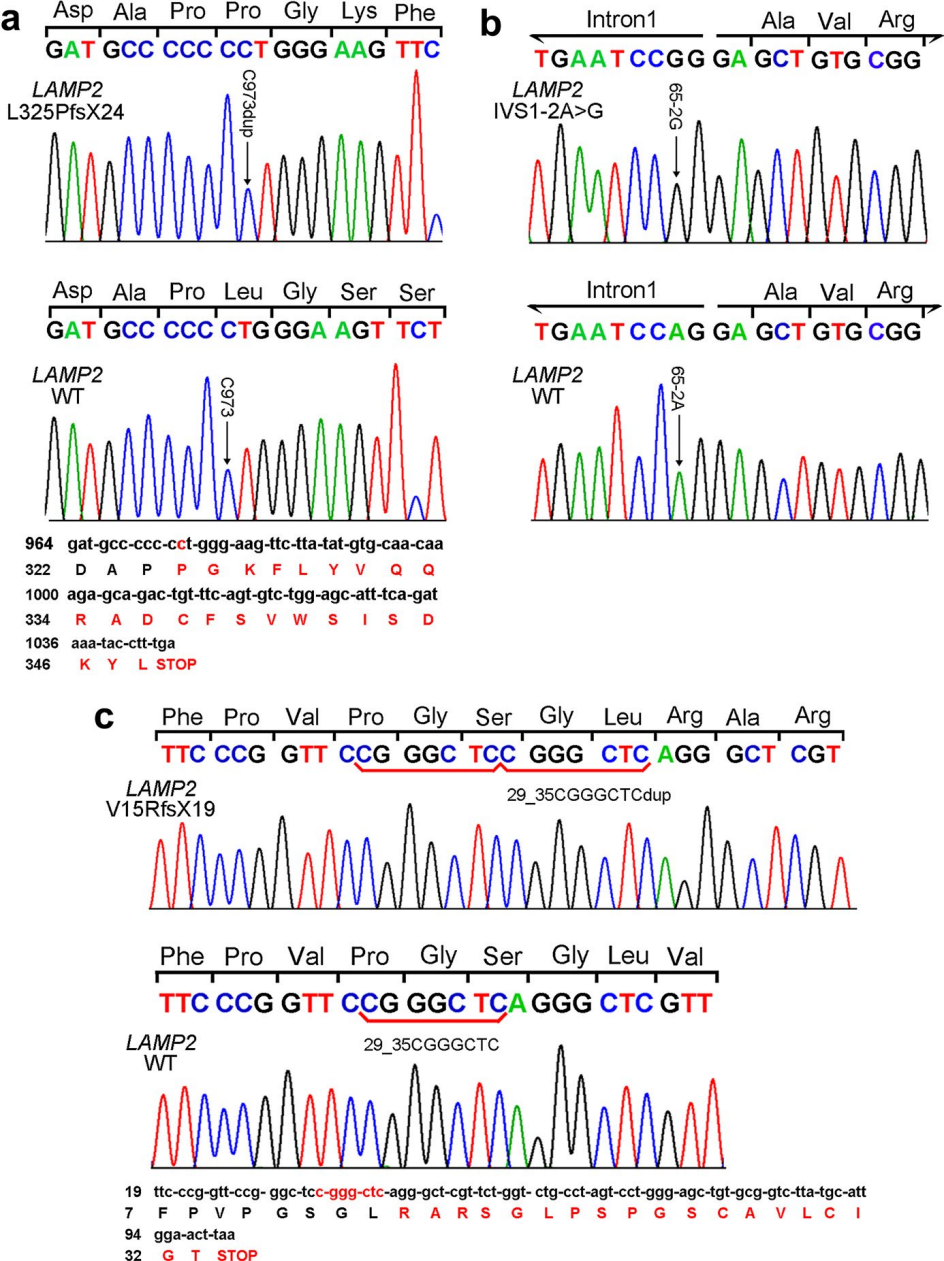
Sanger sequencing validated three *LAMP2* mutations. (a) DNA chromatogram shows a 1‐bp duplication (c. 973dupC) of exon 8 in patient 1, resulting in a frameshift mutation and a premature stop code after introducing 24 extra amino acids (p. L325PfsX24). (b) DNA chromatogram shows an A‐to‐G substitution in the splice‐acceptor site of intron 1 in patient 2(IVS 1‐2A > G). (c) DNA chromatogram shows a 7‐bp duplication (c. 29–35dupCGGGCTC) of exon 1 in patient 3, leading in a novel frameshift mutation and a premature stop codon after introducing 19 extra amino acids (p. V15RfsX19). WT: wild type. Nomenclature in reference to GenBank Accession NM_002294.2

Two frameshift mutations and one splicing mutation of *LAMP2* gene were identified in three patients (Figure 2). In patient 1, a 1‐bp duplication in exon 8 (c. 973dupC) was found, leading to a frameshift mutation and a putatively truncated protein (p. L325PfsX24). In patient 2, there was an A‐to‐G substitution in the splice‐acceptor site of intron 1 (IVS 1‐2A > G), being predicted to delete exon 2 residues and cause a frameshift (Arad et al., 2005; Cetin et al., 2016). In patient 3, a 7‐bp duplication (c. 29‐35dupCGGGCTC) in exon 1, which resulted in a novel frameshift mutation and a putatively truncated protein (p. V15RfsX19).

### 4.3 Histological and immunofluorescent analysis

**Figure 3:**
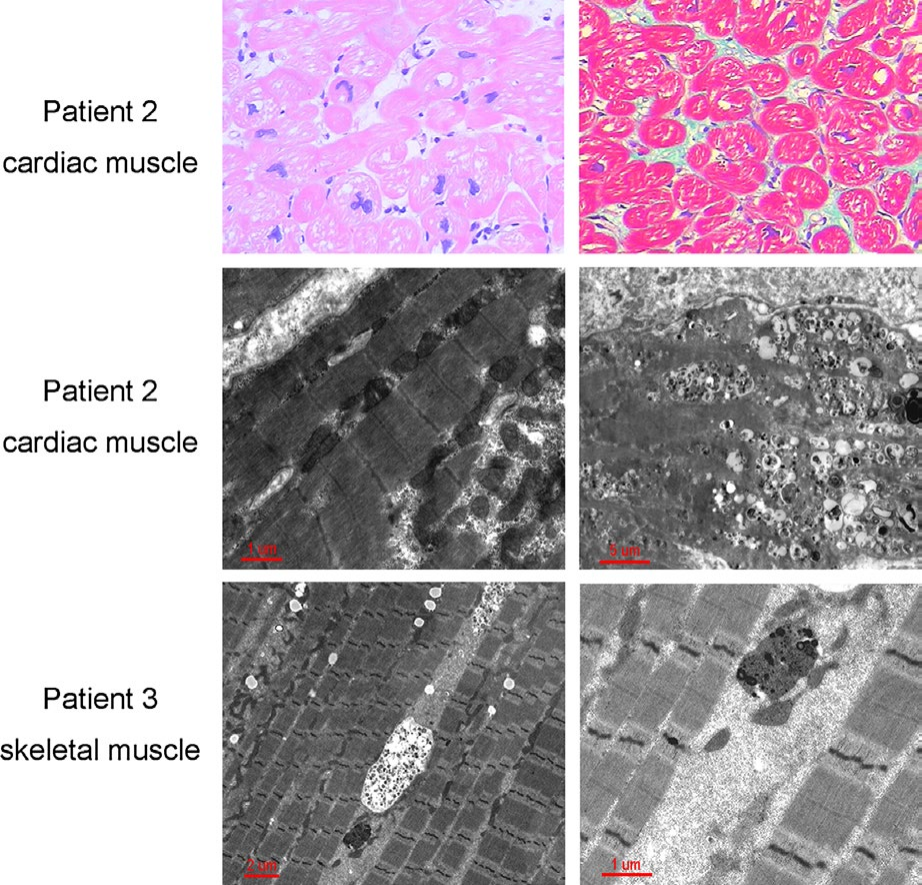
Histopathological analysis of endomyocardial biopsy and skeletal muscle biopsy from patients with Danon patients. Upper panels: Hematoxylin and eosin staining reveals hypertrophied myocytes with profound intracellular vacuolation (left panel, original magnification × 200); Masson's trichrome staining reveals mild interstitial fibrosis with blue color (right panel, original magnification × 200). Middle and lower panels: Electron microscopy shows myofibrillar disruption and intracytoplasmic vacuoles containing autophagicmaterial and glycogen

**Figure 4:**
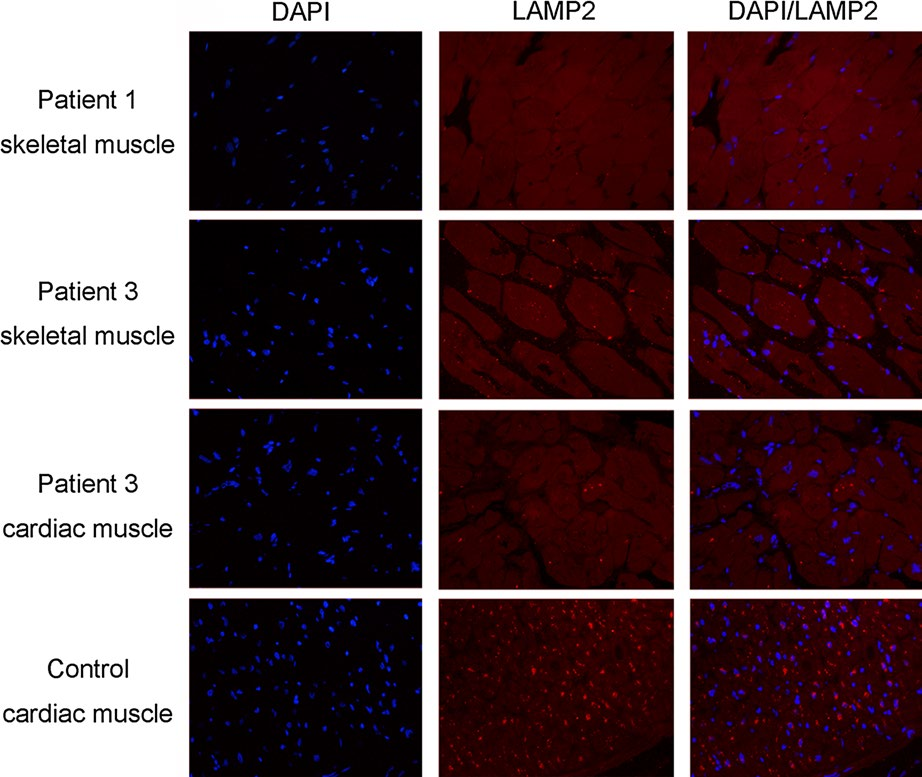
Immunofluorescent analysis of endomyocardial biopsy and skeletal muscle biopsy from patients with Danon patients. Immunofluorescence staining with *LAMP2* antibody demonstrates complete absence of *LAMP2 *protein (red) in the skeletal muscle samples of patient 1 and severe decrease in both skeletal and cardiac muscle samples of patient 3

Skeletal muscle biopsy and endomyocardial biopsy were both available in patient 1 and patient 3, while only endomyocardial biopsy was performed in patient 2. Light microscopy evaluation of the three patients’ cardiac tissue revealed significant enlargement of myocytes with profound intracellular vacuolation, absence of myofiber disarray, and mild or no interstitial fibrosis (upper panels in Figure 3). Electron microscopy showed myofibrillar disruption and intracytoplasmic vacuoles containing autophagic material and glycogen in both skeletal muscle and endomyocardial tissue (middle and lower panels in Figure 3). Immunofluorescent staining of skeletal muscle sections revealed no detectable *LAMP2 *protein in the sample from patient 1 (Figure 4). Compared to control sections from the heart of an age‐matched auto accident victim, staining with the antibody against *LAMP2* protein showed a severe decrease in expression of this protein in both skeletal and cardiac muscle samples of patient 3 (Figure 4).

## 5 DISCUSSION

In this study, genetic testing of 10 patients with unexplained LVH and preexcited ECG patterns led to the identification of three (30%) individuals with Danon disease caused by different *LAMP2 *mutations, including a previously unreported mutation (p. V15RfsX19). Deficiencies of *LAMP2* protein with resultant intracellular vacuoles accumulation in both cardiac and skeletal myocytes were found to account for the development of the disease in our patients. Although Danon disease is considered as a rare entity with unknown prevalence in general population, but cases have been frequently reported in 4% to 33% of selected populations (Arad et al., 2005; Charron et al., 2004; Cheng et al., 2012; Fanin et al., 2006; Fu et al., 2016), in accordance with the finding of our study, suggestive of Danon disease's frequency closely related to the associated clinical phenotypes. To the best of our knowledge, this is the first study reporting the prevalence of Danon disease in patients with coexistent LVH and electrocardiographic preexcitation.

Ventricular preexcitation is a common clinical finding, the majority of which arises from failure of the annulus fibrosus to fully insulate the atrial and ventricular chambers due to the presence of accessory pathways, and also termed Wolff–Parkinson–White (WPW) syndrome if associated with paroxysmal supraventricular tachycardia (Page et al., 2016). It is usually sporadic and of unknown etiology in most cases. In the past two decades, however, a preponderance of evidence suggests a large genetic contribution to this condition when observed in the setting of cardiomyopathy, including mutations in the gene of *LAMP2 *(Liu, Xue, Wu, & Hu, 2016). The reported incidence of electrocardiographic preexcitation of patients with Danon disease is 68.2% and 26.7% in men and women, respectively (Boucek, Jirikowic, & Taylor, 2011). The underlying mechanism responsible for ventricular preexcitation in Danon disease is still confounded by contradictory clinical interpretations. Some studies reported that these individuals had WPW syndrome with catheter ablation required (Bottillo et al., 2016; Boucek et al., 2011; Cheng et al., 2012; Csányi et al., 2016; Samad et al., 2017), but neither electrophysiological details of these pathways nor surface ECGs after ablation were available; others believed that the ECG pattern demonstrating shortened PR interval with QRS slurring was associated with enhanced atrioventricular nodal conduction or “pseudo preexcitation” instead of ventricular preexcitation caused by accessory atrioventricular pathways (Konrad et al., 2017; Lines et al., 2014). However, our recent study proved that ventricular preexcitation in Danon disease was because of the presence of fasciculoventricular pathways characterized by bidirectional conduction and take‐off from the proximal His bundle, rather than the WPW syndrome and enhanced atrioventricular nodal conduction (Liu et al., 2018). Genetic defects in cardiac structural proteins, despite accounting for up to 60% of adolescents and adults with unexplained LVH (Elliott et al., 2014), have not been identified as causative for ventricular preexcitation. In the present study, concurrent ventricular preexcitation in patients with *MYH7*, titin, or *TPM1*variants, therefore, is more likely to be uncommon comorbidity rather than an inherited disorder.

Male predominance typically characterizes Danon disease because of haploinsuffciency of the X‐linked *LAMP2* gene, while affected female patients generally have less severe cardiomyopathy and later onset of symptoms (Boucek et al., 2011). Early identification of Danon disease in male is extremely important for patient care because rapid clinical deterioration leading to cardiac death occurs in young men before 25 years of age if no timely cardiac transplantation considered (Maron et al., 2009). A previous study by Arad et al. suggested that Danon disease is distinguishable from cardiomyopathy due to *PRKAG2* or sarcomere‐protein defects by male sex, early onset of symptoms, massive concentric LVH, asymptomatic elevations of serum‐chemistry values, and electrocardiographic abnormalities, particularly ventricular preexcitation (Arad et al., 2005). In a study of 36 patients with concentric LVH, Cheng et al. reported that Danon disease should be suspected especially when electrocardiographic preexcitation and/or elevated serum biomarkers is present (Cheng et al., 2012). Our data demonstrated that onset at an early age, neurological involvements, prominent electrocardiographic voltages with broad QRS complexes, marked concentric LVH, and abnormal elevation of serum enzyme levels including creatine kinase, aspartate aminotransferase, and lactate dehydrogenase provided important clues to further distinguish LVH and concurrent ventricular preexcitation associated with *LAMP2* mutations from that due to defects of other disease‐causing genes in male. The peculiar ECG profile (wider QRS complex) observed in our patients with Danon disease can be explained by the fact that severe myocardial hypertrophy, especially significant increase in LVPW thickness, necessitates taking longer for ventricular depolarization, while significant elevation of serum enzyme levels could be contributable to the nature of multisystem involvements of Danon disease.

On the basis of these findings, we would like to stress that the association of LVH with ventricular preexcitation, especially in the male teenager with prominent electrocardiographic voltages, wide QRS complexes, concentric LVH, and extracardiac involvements, should highly raise suspicion of Danon disease.

### 5.1 Limitations

Systematic evaluation was not performed in family members of Danon disease patients. However, increasing the number of affected subjects in a family does not necessarily represent an efficient strategy because of the low penetrance of ventricular preexcitation in female patients.

## 6 CONCLUSIONS

In patients with coexistent LVH and ventricular preexcitation, Danon disease is common and easily distinguished by distinct clinical presentations. Comprehensive assessment of these resemble patients can provide valuable findings for early identification and clinical decision making of patients with Danon disease.

## CONFLICT OF INTEREST

None declared.
