# Supplementary material for: Prevalence and clinical characteristics of Danon disease among patients with left ventricular hypertrophy and concomitant electrocardiographic preexcitation
Source: Mol Genet Genomic Med. 2019 Mar 30;7(5):e638. doi: 10.1002/mgg3.638 (PMC6503070; doi:10.1002/mgg3.638)
Supplement: Supplementary file 1 [file MGG3-7-e638-s001.docx]

**SUPPLEMENTAL MATERIAL**

**Supplemental Figure**


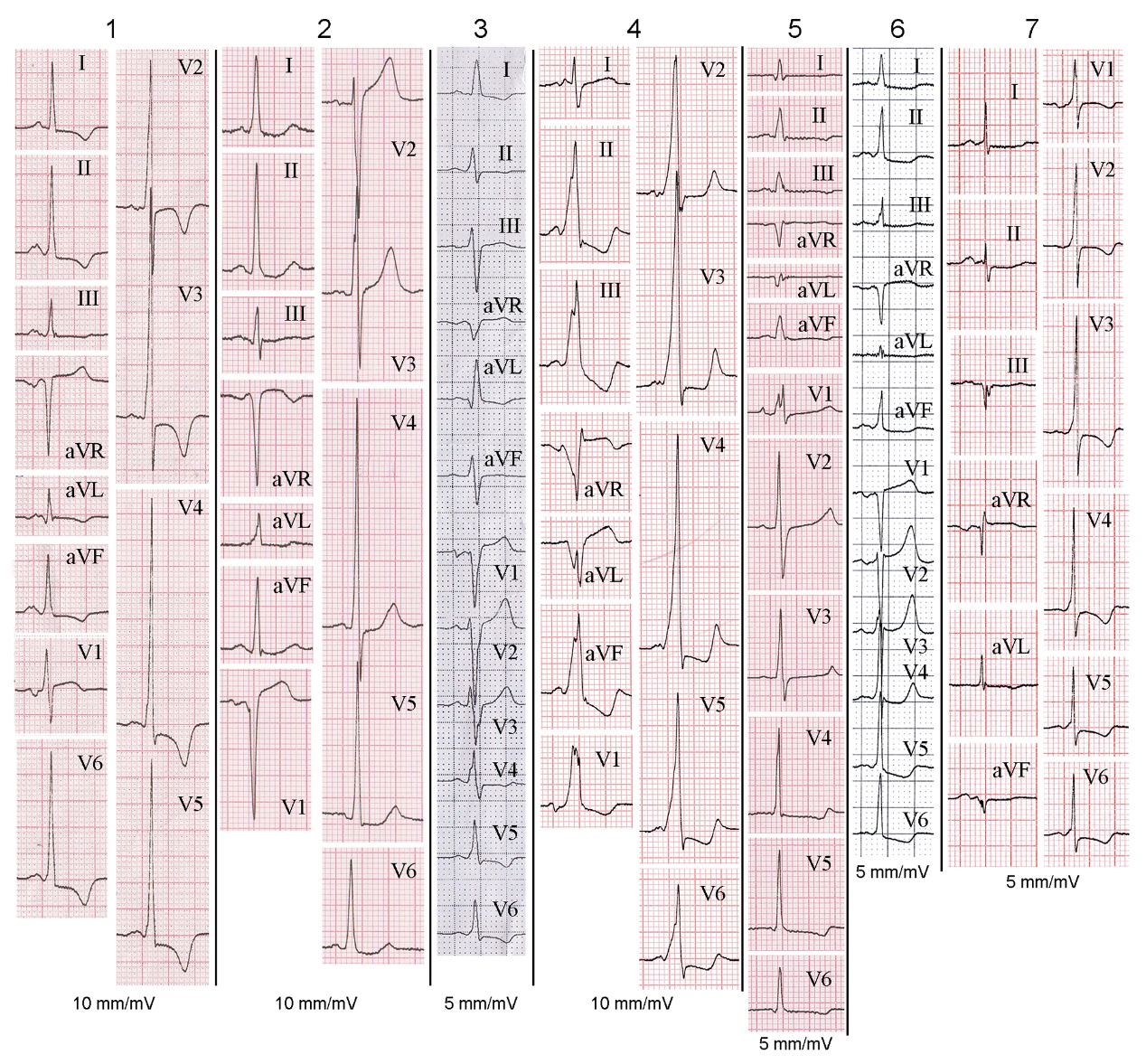


**Supplemental Figure.** Surface ECGs of patients without Danon disease. The case 5 was diagnosed with Fabry’s disease by genetic testing.
