# Supplementary material for: Prevalence and clinical characteristics of Danon disease among patients with left ventricular hypertrophy and concomitant electrocardiographic preexcitation
Source: Mol Genet Genomic Med. 2019 Mar 30;7(5):e638. doi: 10.1002/mgg3.638 (PMC6503070; doi:10.1002/mgg3.638)
Supplement: Supplementary file 2 [file MGG3-7-e638-s002.docx]

**Supplemental table. Clinical characteristics of patients with unexplained LVH**

| Subjects (n = 197) | | Normal range |
| --- | --- | --- |
| Age at diagnosis, years | 54±16 |  |
| Male gender | 122(62%) |  |
| Echocardigraphy |  |  |
| LA size, mm | 42 ± 7 |  |
| LV size, mm | 44 ± 6 |  |
| Maximal IVS thickness, mm | 19 ± 5 |  |
| Maximal LVPW thickness, mm | 13 ± 4 |  |
| Asymmetric hypertrophy | 142 (72%) |  |
| LVEF, % | 65 ± 11 |  |
| LVOTO | 74 (31%) |  |
| NT-proBNP> 3 times of ULN | 149 (76%) † | 0 - 125 pg/ml |
| Serum enzymes |  |  |
| CK> 3-fold ULN | 6 (3%) | 38 – 174 U/L |
| CK-MB> 3-fold ULN | 3 (1.5%) | 0 – 24 U/L |
| AST> 3-fold ULN | 8 (4%) | 15 – 40 U/L |
| ALT> 3-fold ULN | 4 (2%) | 9 – 50 U/L |
| LDH> 3-fold ULN | 5 (2.6%) † | 109 – 245 U/L |
| cTnT> 3-fold ULN | 51 (26%) † | 0 – 14 pg/ml |

† This value was not available in 1 patient. ALT: alanine aminotransferase; AST: aspartate aminotransferase; CK: creatine kinase; cTnT: cardiac troponin T; IVS: interventricular septum; LA: left atrium; LDH: lactate dehydrogenase; LV: left ventricle; LVEF: left ventricular ejection fraction; LVH: left ventricular hypertrophy; LVOTO: left ventricular outflow tract obstruction; LVPW: left ventricular posterior wall; NT-proBNP: N-terminal pro-brain natriuretic peptide; ULN: upper limit of normal range.
